# Exploring Hydrogen Sources in Catalytic Transfer Hydrogenation: A Review of Unsaturated Compound Reduction

**DOI:** 10.3390/molecules28227541

**Published:** 2023-11-11

**Authors:** Batoul Taleb, Rabih Jahjah, David Cornu, Mikhael Bechelany, Mohamad Al Ajami, Ghenwa Kataya, Akram Hijazi, Mohammad H. El-Dakdouki

**Affiliations:** 1 Platform for Research and Analysis in Environmental Sciences (PRASE), Doctoral School of Science and Technology, Lebanese University, Beirut P.O. Box 6573/14, Lebanon; batoul.taleb96@gmail.com (B.T.); jahjah_r@hotmail.com (R.J.); mohamad.r.alajami@hotmail.com (M.A.A.); ghenwa.kataya.1@ul.edu.lb (G.K.); akram.hijazi@ul.edu.lb (A.H.); 2 Department of Chemistry, Faculty of Science, Beirut Arab University, Debbieh P.O. Box 11-5020, Lebanon; 3 Institut Européen des Membranes (IEM), UMR 5635, University of Montpellier, ENSCM, CNRS, Place Eugène Bataillon, 34095 Montpellier, France; david.cornu@umontpellier.fr; 4 Applied Mathematics and Bioinformatics (CAMB), Gulf University for Science and Technology (GUST), Mubarak Al-Abdullah 32093, Kuwait

**Keywords:** hydrogen transfer, hydrogen donor molecules, alcohol, formic acid, amine, glycerol, 1,4-cyclohexadiene

## Abstract

Catalytic transfer hydrogenation has emerged as a pivotal chemical process with transformative potential in various industries. This review highlights the significance of catalytic transfer hydrogenation, a reaction that facilitates the transfer of hydrogen from one molecule to another, using a distinct molecule as the hydrogen source in the presence of a catalyst. Unlike conventional direct hydrogenation, catalytic transfer hydrogenation offers numerous advantages, such as enhanced safety, cost-effective hydrogen donors, byproduct recyclability, catalyst accessibility, and the potential for catalytic asymmetric transfer hydrogenation, particularly with chiral ligands. Moreover, the diverse range of hydrogen donor molecules utilized in this reaction have been explored, shedding light on their unique properties and their impact on catalytic systems and the mechanism elucidation of some reactions. Alcohols such as methanol and isopropanol are prominent hydrogen donors, demonstrating remarkable efficacy in various reductions. Formic acid offers irreversible hydrogenation, preventing the occurrence of reverse reactions, and is extensively utilized in chiral compound synthesis. Unconventional donors such as 1,4-cyclohexadiene and glycerol have shown a good efficiency in reducing unsaturated compounds, with glycerol additionally serving as a green solvent in some transformations. The compatibility of these donors with various catalysts, substrates, and reaction conditions were all discussed. Furthermore, this paper outlines future trends which include the utilization of biomass-derived hydrogen donors, the exploration of hydrogen storage materials such as metal-organic frameworks (MOFs), catalyst development for enhanced activity and recyclability, and the utilization of eco-friendly solvents such as glycerol and ionic liquids. Innovative heating methods, diverse base materials, and continued research into catalyst-hydrogen donor interactions are aimed to shape the future of catalytic transfer hydrogenation, enhancing its selectivity and efficiency across various industries and applications.

## 1. Introduction

Hydrogenation is a chemical reaction between an unsaturated molecule and a hydrogen molecule, usually in the presence of a catalyst. This reaction is crucial, especially in industry, because it is used to reduce or saturate organic compounds. It has many important uses, such as converting vegetable oils into solid fats, as well as in pharmaceuticals synthesis, plastic manufacturing, and producing chemicals from petroleum [1].

There have been two distinct methods employed to conduct hydrogenation in this study: direct hydrogenation, which utilizes pressurized hydrogen gas, and transfer hydrogenation. Transfer hydrogenation involves the transfer of hydrogen from one molecule to another using a hydrogen donor other than molecular hydrogen.

While direct hydrogenation has been the conventional method used across various sectors, transfer hydrogenation has emerged as an attractive alternative to this approach, and it has recently become the center of research in hydrogenation science. The significance of transfer hydrogenation can be attributed into several key points: (i) Enhanced safety measures, as this method eliminates the need for potentially hazardous pressurized H_2_ gas; (ii) Availability of cost-effective and easily manageable hydrogen donors; (iii) The ability to recycle the primary byproduct; (iv) Accessibility and stability of the catalysts used; (v) The potential for catalytic asymmetric transfer hydrogenation in the presence of chiral ligands [2]. 

Transfer hydrogenation was first observed in 1925 by Meerwein–Ponndorf–Verley (MPV). They utilized alcohols as the source of hydrogen (H-source) and aluminum alkoxide as a homogeneously acting catalyst to facilitate the reduction of carbonyl compounds [3,4]. Based on this work, researchers have extended the MPV reduction to carbonyl compounds using metals such as aluminum, zirconium, lanthanum, cerium, samarium, and ytterbium [5].

**Scheme 1:**
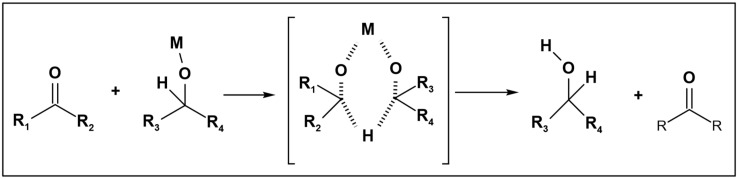
Hydrogen transfer in the MPV reduction via a cyclic transition state (Wang & Astruc, 2015) [8].

In a study aimed at understanding the mechanism of the homogeneous MPV reduction, the authors proposed that both the alcohol, which acts as the reducing agent, and the carbonyl compound bind to the same metal center, forming a six-membered cyclic transition state (Scheme 1) [6,7,8]. Interestingly, the reverse of this reaction, referred to as oxidation, was also examined and studied by Oppenauer [9]. 

The MPV reduction technique is widely used in both the academic and industrial sectors, particularly in the production of flavor compounds, due to its high efficiency in reducing the keto functional group under mild conditions [8]. Despite these advantages, MPV has its drawbacks: it needs significant amounts of reagents and side reactions may occur, which affects the yield and selectivity of desired products. In addition, MVP is sensitive to moisture, which can be problematic, especially with an aluminum catalyst [10].

The next significant advancement in transfer hydrogenation was the utilization of late transition metals as catalysts, exceeding the turnover frequency reached with MPV catalysts. In the 1970s, Sasson et al. studied the transfer hydrogenation of α,β-unsaturated ketones using [RuCl_2_(PPh_3_)_3_] as a catalyst and 1-phenylethanol as a hydrogen source [11]. This discovery prompted further in-depth investigations into diverse aspects of transfer hydrogenation reactions.

Chowdhury et al. found that the catalytic activity of the [RuCl_2_(PPh_3_)_3_] catalyst increased by 10^3^–10^4^ times when a small amount of NaOH was added [12]. The use of bases increased the catalytic activity by deprotonating the substrate and promoting its complexation with the metal ion [13].

A notable achievement in hydrogenation is the development of asymmetric transfer hydrogenation, considered an important branch of transfer hydrogenation, especially within the fragrance and pharmaceutical sectors [14]. It leads to the production of a wide variety of chiral molecules by performing a selective hydrogenation of unsaturated compounds [15].

While remarkable progress has been made in developing both symmetric and asymmetric transfer hydrogenation using late transition metals, a significant shift has occurred toward the utilization of heterogeneous catalysts. Heterogeneous catalysis presents a solution to the challenges associated with homogeneous catalysis such as catalyst separation, recovery, and reuse, making it an interesting approach for researchers. A diverse range of metal catalysts (nickel, palladium, gold, etc.) and catalytic supports (carbon, alumina, titanium, etc.) [16] have been tested and have shown excellent efficiency in catalytic transfer hydrogenation reactions, especially in performing the selective reduction of various unsaturated compounds, from carbonyl compounds to alkyne and alkene.

**Figure 1:**
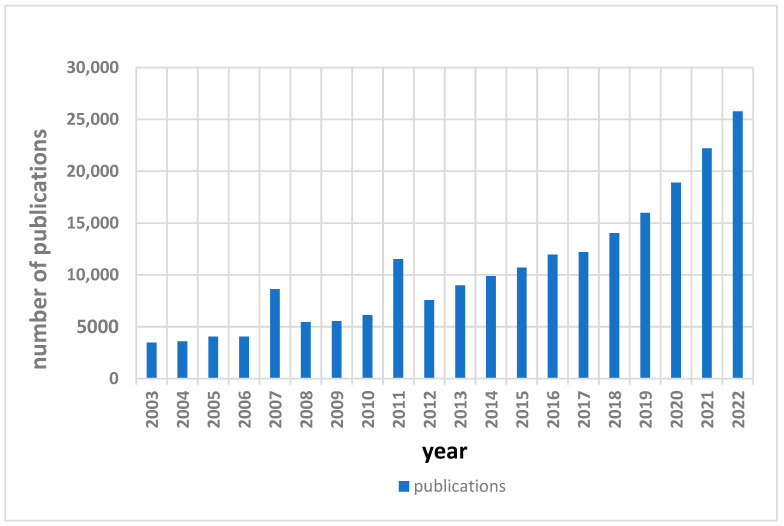
Number of publications on transfer hydrogenation reactions (2003–2022, data source: dimensions.ai, accessed on 6 November 2023).

In recent years, catalytic transfer hydrogenation has garnered significant attention in the realm of diverse chemical reactions. According to data from the dimensions.ai website, the number of publications in the last two decades has reached 286,603 papers, underscoring the high importance of this topic (Figure 1). Its significance lies in providing efficient alternatives to conventional hydrogenation methods, characterized by improved safety and cost-effectiveness, as well as broader applicability across various chemical transformations. While numerous comprehensive reviews have examined the catalysts involved [2,17,18], this review takes a distinctive approach by focusing on a critical aspect of a reaction that has received relatively less investigation: hydrogen donor molecules.

Hydrogen donor molecules, which encompass a variety of compounds such as alcohols, water, and formic acid, and unconventional donors, such as glycerol, are pivotal components of catalytic transfer hydrogenation. Our review explores their unique properties, reactivity, and potential applications. Additionally, we delve into the catalytic mechanisms of various hydrogenation systems, shedding light on how the choice of a hydrogen donor can significantly impact the outcomes of the reaction, affecting product formation, yield, and conversion percentages.

Furthermore, this review looks ahead and outlines potential future trends that could shape the context of catalytic transfer hydrogenation. These include the possible exploration of hydrogen donors derived from biomass, the investigation of innovative hydrogen storage materials, and the consideration of advanced catalysts with enhanced sustainability and selectivity. Additionally, it highlights the potential for future studies to examine environmentally friendly solvents and novel heating techniques in the context of catalytic transfer hydrogenation. Our aim from this review is to offer a comprehensive resource that not only highlights the present state of the field but also offers glimpses into potential directions for future research. Through these efforts, we hope to contribute to the continued advancement of catalytic transfer hydrogenation and its potential for sustainable and efficient chemical transformations.

## 2. Hydrogen Donors

As mentioned above, a hydrogen source other than molecular hydrogen is needed in hydrogen transfer reactions. For the hydrogen to transfer from the donor molecule to the unsaturated compound, the hydrogen donor must satisfy two main conditions: (1) it should have a low oxidation potential, allowing hydrogen abstraction to occur in the presence of a catalyst under mild conditions; (2) it should have the capacity to loosely bind to the catalytic center [19].

There are many compounds that can act as hydrogen donors; the choice of which to use depends on a few important parameters: (1) The type of catalyst being used, whether it is MPVO or a transition metal catalyst; (2) Whether it can work well under mild conditions; (3) The nature of the functional group to be saturated; (4) The influence of the donor molecule on the equilibrium of the reaction; (5) Whether the donor molecule helps in avoiding the formation of any undesirable byproducts [20].

### 2.1. Alcohol Hydrogen Donor

**Scheme 2:**
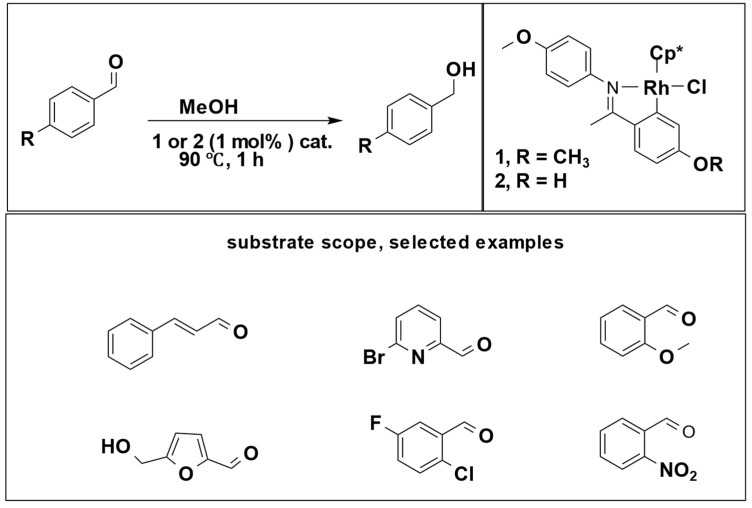
Transfer hydrogenation of benzaldehyde, with methanol as the hydrogen donor, in the presence of a rhodium catalyst (Aboo et al., 2018) [21].

Alcohols are frequently employed as hydrogen donors, particularly in reduction reactions utilizing the Meerwein–Ponndorf–Verley (MPV) catalyst [5]. In this context, only alcohol molecules serve as hydrogen donors, with both primary and secondary alcohols being utilized. Jainling et al. utilized methanol as a hydrogen donor in the presence of a rhodium catalyst to carry out the hydrogenation of benzaldehyde. This process achieved a substantial yield percentage and chemoselectivity, as illustrated in (Scheme 2) [21].

**Scheme 3:**
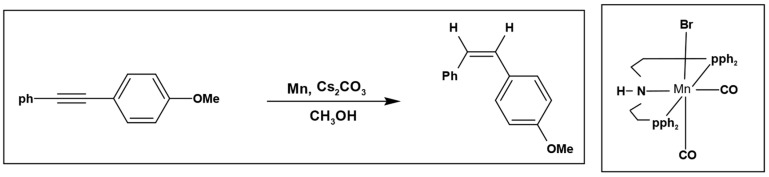
Reduction of alkynes catalyzed using manganese pincer complexes in the presence of methanol used as a hydrogen donor (Sklyaruk et al., 2020) [22].

Furthermore, Magnus et al. used methanol as a hydrogen source to perform selective hydrogenation of alkynes with manganese pincer complex as a catalyst (Scheme 3) [22].

The term “pincer” refers to a ligand type that occupies adjacent binding sites in a metal complex and typically adopts a meridional arrangement. Pincer ligands gained significant attention from scientists in the 1990s and have become a subject of thorough investigation, as evidenced by the increasing number of review articles focusing on transition metal complexes with pincer ligands [23,24]. The significance of these ligands lies in their distinct combination of properties. They hold the donor groups in a predictable arrangement which allows them to control the nature of the coordination sphere. In addition, they are highly effective for catalyzing asymmetric reactions. They also exhibit remarkable thermal stability, making them suitable for homogeneous catalysis [25].

Hansjörg Grützmacher et al. revealed that ethanol was an excellent hydrogen donor compared to methanol and 2-propanol in the hydrogenation of carbonyl compounds using the rhodium catalyst [26]. Johannes G. de Vries et al. successfully conducted a base-free transfer hydrogenation of esters with iron catalysis, employing ethanol as the hydrogen supplier [27]. The reaction equation and the hydrogen donors investigated are presented in (Table 1). In this reaction, methyl benzoate (a) serves as the starting material, undergoing hydrogenation with an iron catalyst to yield benzyl alcohol (b), as the main product, and a side product (c), resulting from a transesterification reaction. The highest conversion (>99%) and percentage yield (88%) of the main product (alcohol) were achieved using ethanol. This research work [26] stands among the limited number of conducted studies. This is attributed to the challenge that esters are less readily reduced compared to ketones [28]. Additionally, the transfer hydrogenation reaction occurred without the presence of a base additive, which has been shown to be an essential element [29,30].

While ethanol has shown promise in certain reactions, its systematic exploration as a hydrogen source in transfer hydrogenation has been limited. This is mainly because ethanol often hinders the catalyst’s performance by forming stable and inactive carbonyl complexes [31,32]. It has been noted that secondary alcohols are more efficient due to their sigma inductive effects. Further, 2-propanol is, notably, the primary choice among various secondary alcohols for use as a hydrogen donor. This is because 2-propanol possesses several favorable properties, including widespread availability, affordability, a suitable boiling point, and solubility. Moreover, the acetone produced as a result of 2-propanol oxidation is non-toxic and can be easily separated from the reaction mixture [33].

**Scheme 4:**
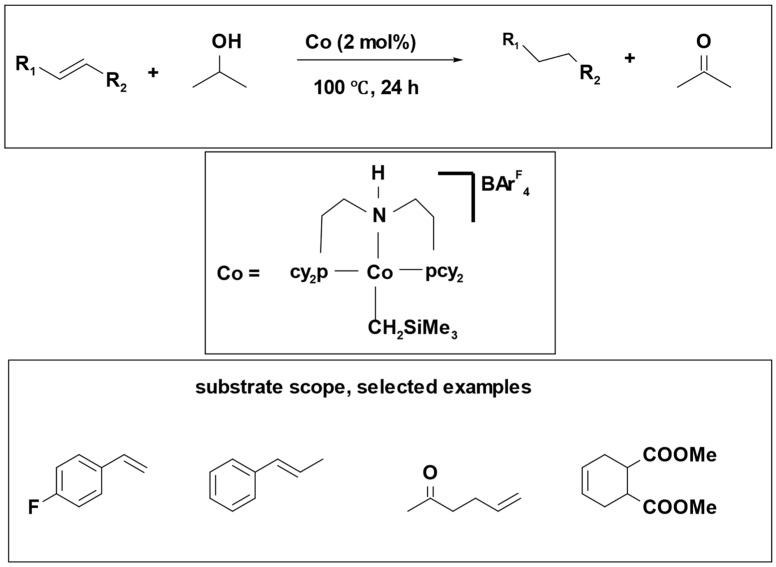
Reduction of alkenes using a cobalt pincer complex as a catalyst in the presence of isopropanol as a hydrogen donor (Zhang et al., 2016) [34].

Catalytic transfer hydrogenation of olefins was successfully accomplished using isopropanol as a hydrogen source. This process employed cobalt (II) pincer ligand complex which proved effective for hydrogenating different kinds of olefins, including aromatic, aliphatic, terminal, and internal alkenes. The yields obtained from this method ranged from good to excellent [34] (Scheme 4). Notably, both the chosen catalyst and the hydrogen donor demonstrated a high degree of compatibility with various functional groups, rendering them well-suited for olefin transfer hydrogenation reactions.

**Scheme 5:**
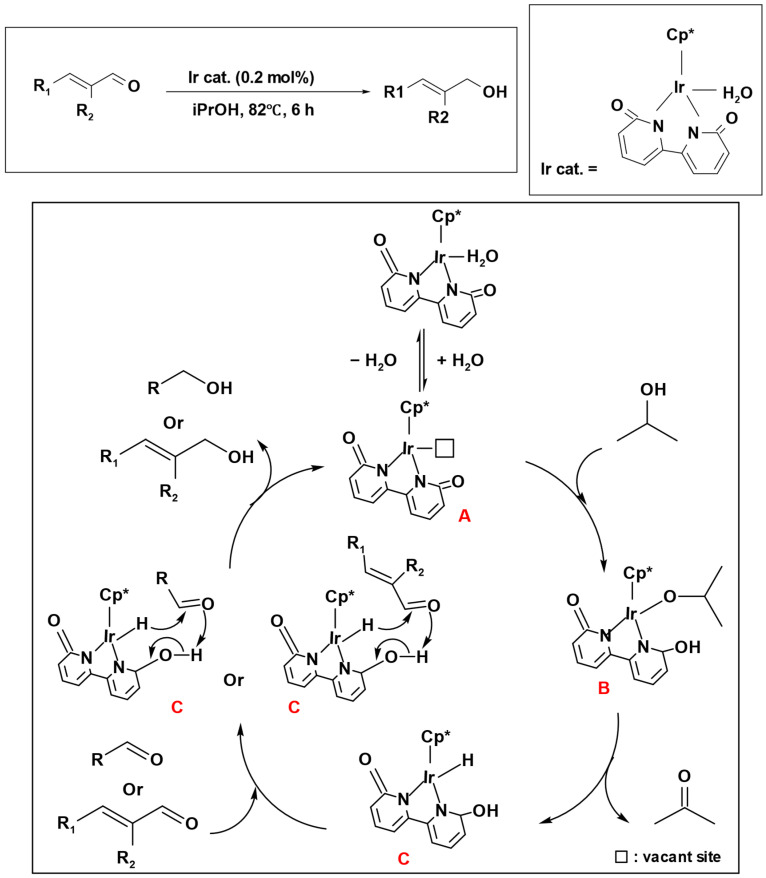
Transfer hydrogenation reaction of unsaturated aldehyde using iridium as a catalyst and isopropanol as a hydrogen donor (Wang et al., 2018) [35].

Fengi Li et al. made a significant work regarding the excellent efficiency of an iridium bifunctional catalyst in selectively transferring hydrogen to unsaturated aldehydes using isopropanol under neutral conditions (Scheme 5) [35].

The initial step involves the removal of H_2_O to generate the unsaturated species **A** (a 16-electron complex) holding a 2,2′-bipyridonate ligand [36]. This ligand accepts a proton from isopropanol, leading to the formation of alkoxy iridium species **B**. This species then undergoes a β-elimination reaction, producing iridium hydride species **C** and acetone. The unsaturated compound (aldehyde) is inserted and the transfer hydrogenation process takes place. This mechanism involves the transference of a hydride from iridium and the hydroxy proton from the bipyridine ligand of complex **C** to the carbonyl group of the aldehyde, ultimately leading to the production of the desired saturated alcohol product.

Homogeneous catalysts have traditionally been favored for their exceptional selectivity. Moreover, they display remarkable activity, particularly in asymmetric catalysis. However, these advantages are counterbalanced by their significant cost and the challenges associated with their separation and reuse. This prompted a shift in research focus towards heterogeneous catalysts, which are cost-effective and easily separable [37].

**Scheme 6:**
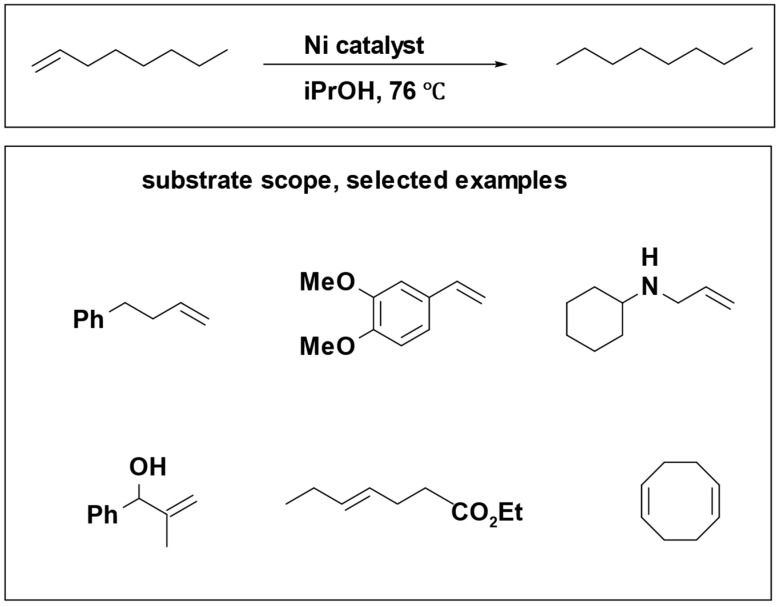
Nickel-catalyzed hydrogen transfer of alkenes using isopropanol as a hydrogen donor (Alonso et al., 2009) [37].

Francisco Alonso et al. conducted catalytic transfer hydrogenation experiments on various functionalized and non-functionalized olefins. They utilized nickel nanoparticles as the catalyst and 2-propanol as the source of hydrogen. This heterogeneous catalyst efficiently catalyzed the reaction, resulting in high yields and chemoselectivity for specific substrates [38] (Scheme 6). In fact, the efficiency of the nickel catalyst has been demonstrated in various transfer hydrogenation reactions, including the α-alkylation of methyl ketones with primary alcohols [39,40] and the transfer hydrogenation of carbonyl compounds [41,42].

**Scheme 7:**
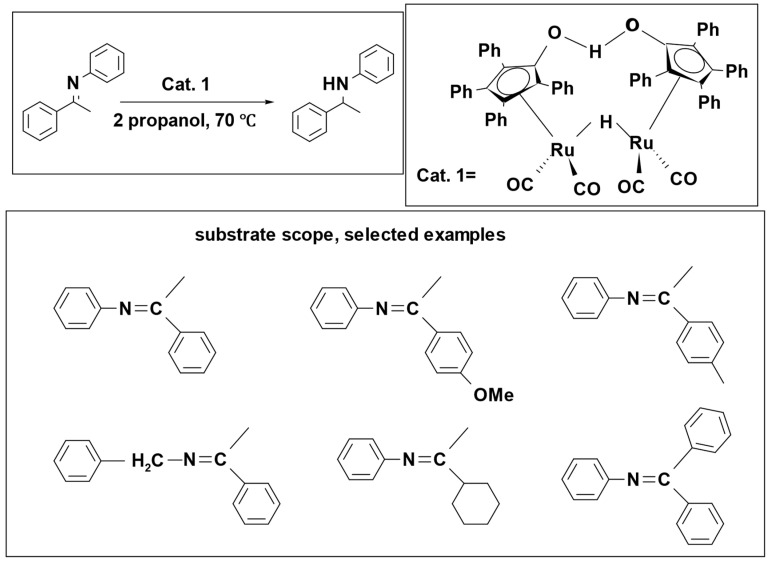
Asymmetric transfer hydrogenation of imines using a rhodium catalyst and isopropanol as a hydrogen donor (Samec & Bäckvall, 2002) [48].

While the transfer hydrogenation reaction of carbonyl compounds has been extensively investigated in the past two decades [35,43,44,45,46], the corresponding reaction involving imines has received less attention, although it holds significant importance for the synthesis of pharmaceuticals and agrochemicals. Jan E. Bäckvall et al. explored the transfer hydrogenation of imines. They utilized [RuCl_2_(PPh_3_)_3_] as the catalyst, 2-propanol as the hydrogen donor, and K_2_CO_3_ as a base [47]. In a further investigation, their focus shifted to the asymmetric transfer hydrogenation of imines. For this purpose, they employed a dimeric rhodium catalyst and isopropanol as the source of hydrogen (Scheme 7) [48]. In this process, catalyst **1** broke down into two components, species **2** and **3**. Species **2** was responsible for hydrogenation, while species **3** was involved in dehydrogenation [49]. In the reaction, species **2** hydrogenated the imine to form an amine, and, simultaneously, species **3** caused the dehydrogenation of 2-propanol, resulting in the formation of acetone. Isopropanol effectively served as a hydrogen donor, demonstrating its significant impact as a solvent. The reaction achieved remarkable success, resulting in a percentage yield exceeding 97%.

The catalytic transfer hydrogenation of furfural to furfuryl alcohol has attracted significant interest, mainly because furfural can be converted into a diverse array of chemicals and liquid transportation fuels [50]. Moreover, furfuryl alcohol has the potential to be a precursor for various valuable chemicals, including tetrahydrofurfuryl alcohol, and has various industrial applications [51].

The research attention has also turned to nitrogen-doped carbon-supported iron catalysts. These catalysts exhibit the ability to selectively hydrogenate compounds, such as nitroarenes, to anilines using molecular hydrogen (H_2_) or alternative hydrogen donors, such as formic acid and hydrazine hydrate [52,53].

In a study merging these interests, Jiang Li et al. utilized nitrogen-doped carbon-supported iron catalysts for the catalytic transfer hydrogenation of furfural to furfuryl alcohol. Optimal outcomes were achieved by employing 50 mg of Fe-L1/C-800 catalyst at 120 °C, with 2-butanol as the hydrogen source [54]. When testing primary alcohols such as methanol, ethanol, and 1-propanol, it was observed that they led to low selectivity for furfuryl alcohol. Notably, a significant contrast was shown in the effectiveness between 2-propanol and 2-butanol, which could be attributed to the differing alcohol dehydrogenation activities. On the other hand, cyclohexanol exhibited lower selectivity due to its relatively high viscosity. As a result, 2-butanol was the preferred choice for the hydrogen donor (Table 2).

Because this reaction holds great significance, numerous research efforts have focused on catalytic transfer hydrogenation of furfural [55] using various alcohol hydrogen donors. Some of these studies are outlined in (Table 3).

Alcohols such as benzyl alcohol [66], 1-butanol [67], and glycerol [68] have also been identified as effective hydrogen sources in catalytic transfer hydrogenation reactions.

### 2.2. Water

Though the use of water as a hydrogen source has been relatively limited, it holds great promise due to: (1) Being safe and eco-friendly; (2) Its affordability; (3) The ease of tracking reactions through isotopic labeling with D_2_O. However, there is a challenge when it comes to water-mediated reactions catalyzed by transition metals. These reactions result in the formation of an organometallic intermediate that does not readily mix with water. To address this issue, a reductant is utilized. Common metals such as lithium, sodium, and zinc can act as reducing agents for hydrogenating carbon-carbon π-systems, even in the presence of protic reagents such as amines, alcohols, acids, and water [69].

**Scheme 8:**
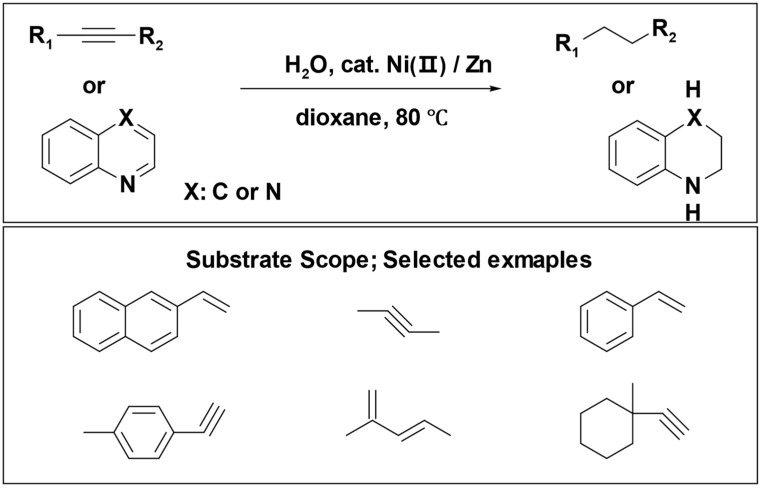
Transfer hydrogenation of alkenes and alkynes using nickel as a catalyst and water as the hydrogen source (Hu et al., 2019) [70].

Yuanhong Liu et al. introduced an innovative approach for the transfer hydrogenation of alkenes and alkynes, employing water as the hydrogen source. This reaction system utilized nickel (II) salt as the pre-catalyst and zinc powder as the reducing agent. Notably, this reaction occurred under mild conditions, accommodating a broad range of substrates and demonstrating tolerance for diverse functional groups (Scheme 8) [70].

Yoshio Inoue et al. carried out a rhodium-catalyzed hydrogenation of olefins, including α,β-unsaturated ketones. They used H_2_O or D_2_O as the hydrogen source and zinc metal as the reducing agent. Various olefin substrates were tested. Terminal olefins (aromatic and aliphatic) produced fully saturated products in high yields. In contrast, internal olefins resulted in partially reduced products with moderate yields. Interestingly, α,β-unsaturated ketones yielded selective 1,4-reduced products, with no alcohol product observed [69].

**Scheme 9:**
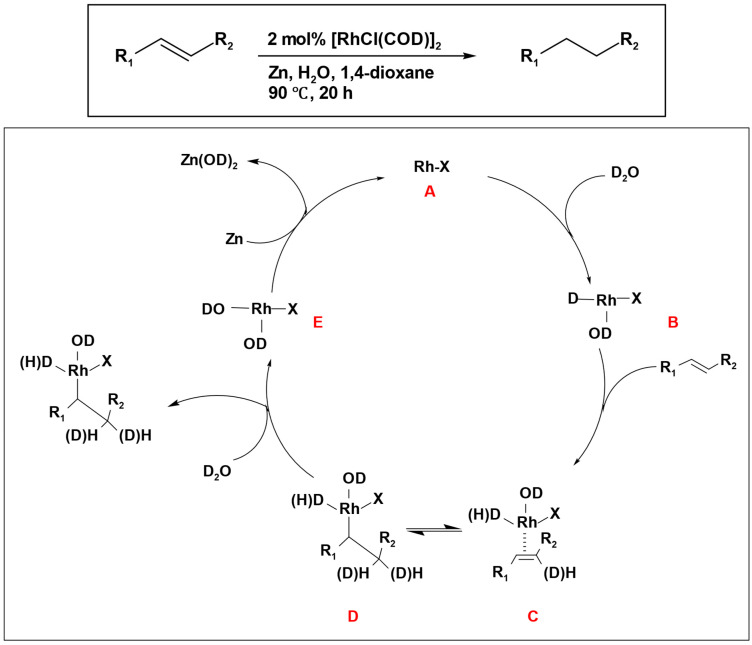
Rhodium-catalyzed hydrogenation of olefins using water as the hydrogen source (Sato et al., 2006) [69].

Deuterium-labeling experiments showed that, when deuterium was used in the reduction of simple olefins such as styrene, a combination of mono-, di-, and tri-deuterated products emerged as a result of a deuterium scrambling process. This prompted the proposal of a mechanism that encompasses a trivalent mono-deuterio-rhodium intermediate participating in addition to a carbon-carbon double bond (Scheme 9). The process started with an oxidative addition of D_2_O, resulting in the formation of a trivalent mono-deuterio-rhodium intermediate (**B**). Subsequently, the alkene compound was introduced, leading to the formation of complex (**C**). Through a migratory insertion reaction, this complex transformed into (**D**). In this step, the reversible formation of the complex **C** caused the scrambling of deuterium. The hydration of complex **D** by D_2_O, followed by reductive elimination, led to the formation of the desired product (alkane) and a trivalent deuterio-rhodium complex **E**. In the end, the reduction of **E** by zinc metal resulted in the regeneration of the monovalent rhodium complex **A** (Scheme 9).

**Scheme 10:**
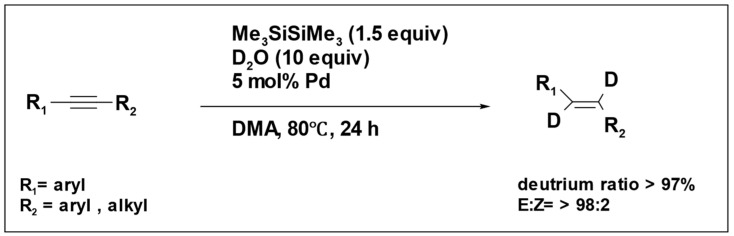
Palladium-catalyzed reduction of alkynes (Shirakawa et al., 2005) [71].

Shirakawa et al. successfully reduced alkynes, producing *E*-1,2-di-deuterioalkenes with good selectivity (*E*:*Z* > 98:2). Their approach involved the use of D_2_O as the deuterium source, hexamethyldisilane as the reducing agent, and a palladium catalyst (Scheme 10) [71].

The reduction of alkynes with Me_3_SiSiMe_3_–D_2_O follows a similar catalytic cycle to the one proposed by Trost for palladium-catalyzed alkyne reduction using hydrosilane and acetic acid [72]. Numerous studies have focused on reducing both alkenes and alkynes using water as the hydrogen source. More recently, several studies have utilized water in combination with reducing agents such as magnesium [73], Cp_2_TiC [74], B_2_(OH)_4_ [75], and B_2_Pin_2_ [76].

### 2.3. Formic Acid and Its Salts

Many studies have reported a chemical challenge encountered in the asymmetric transfer hydrogenation of ketones into alcohols when 2-propanol is utilized as the hydrogen source [77,78]. This challenge involves the potential deterioration of the enantiomeric purity of the chiral product. This is due to the occurrence of a reverse process caused by structural similarities between the hydrogen donor and the product, both of which are secondary alcohols [20]. However, when formic acid is employed as the hydrogen donor, it undergoes dehydrogenation to produce carbon dioxide. The irreversible nature of the reaction ensures that the process remains under kinetic control.

It has been reported that adding a weak base such as triethylamine is enough to facilitate hydrogen transfer, enabling the transfer hydrogenation reaction to be conducted under aqueous conditions [79]. Consequently, the combination of formic acid and an amine as a hydrogen source has been shown as a good alternative.

**Figure 2:**
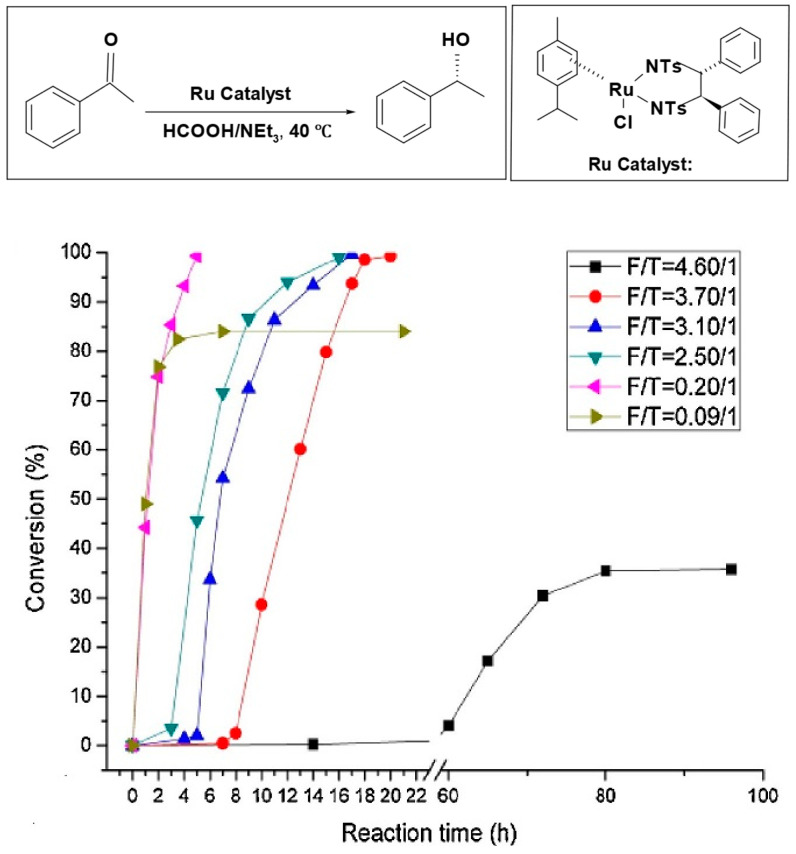
Percentage conversion versus reaction time for the asymmetric transfer hydrogenation (ATH) of acetophenone using a Ru catalyst in F-T mixtures with varying initial F/T ratios. (Reprinted with permission from Ref. [80]. Copyright 2012, Elsevier).

Bolun Yang et al. examined how the ratio of formic acid to amine (F/T) influenced the speed of reduction and the selectivity of asymmetric transfer hydrogenation of ketones, using the Ru-TsDPEN catalyst [80]. When the F/T ratio is high (acidic medium), the reaction needs a long time to reach a high conversion; at (F/T = 4.6/1), the reaction had an extended induction period and reached only 39% conversion after 150 h. On the other hand, at a lower F/T ratio (basic medium), the reaction rate significantly increases; at (F/T = 0.2/1), the reduction started instantaneously and reached a 100% conversion after 5 h with high enantiomeric excess (%*ee*). However, at (F/T = 0.09/1), the reaction reached only 80% conversion due to the limited amount of hydrogen source available relative to the substrate. Based on this result, the optimal F/T molar ratio is 0.2/1 (Figure 2).

**Scheme 11:**
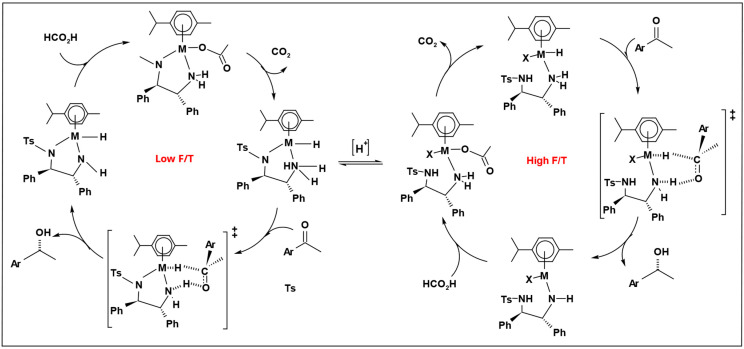
Proposed catalytic cycles for the asymmetric transfer hydrogenation (ATH) of acetophenone under near-neutral conditions (I) and acidic conditions (II) in an F-T solution (Zhou et al., 2012) [80].

Because of the noticeable distinction between high and low F/T ratios, two catalytic cycles were proposed depending on the F/T molar ratios (Scheme 11). The catalytic cycle proceeded through a concerted transfer hydrogenation transition state. At high F/T, ratio protonation occurs at both the hydride and TsDPEN ligands, driving the catalyst into a less active and less enantioselective cycle.

**Figure 3:**
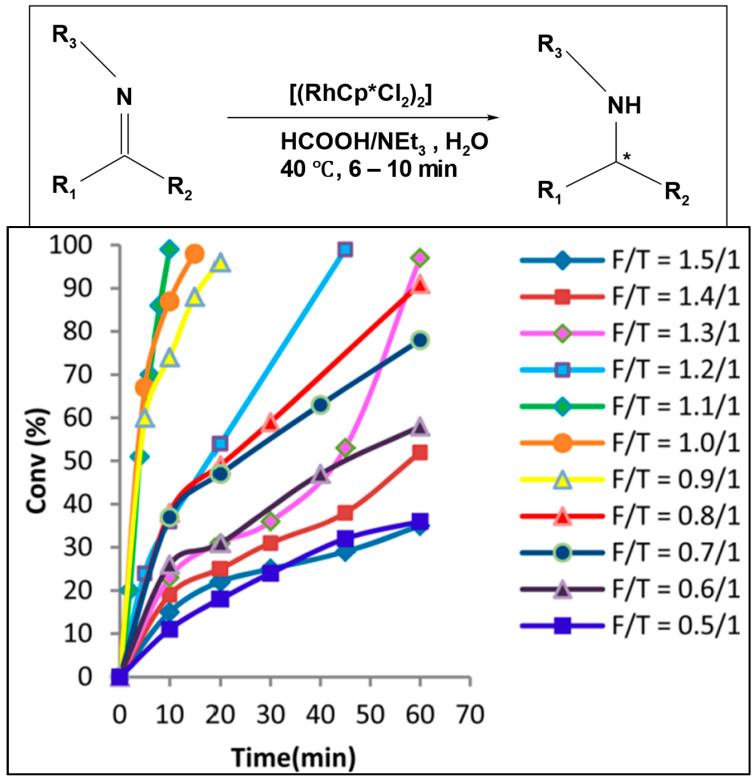
Asymmetric transfer hydrogenation of imines in water in F-T mixtures at different initial F/T ratios. (Reprinted with permission from Ref. [84]. Copyright 2015, American Chemical Society).

Catalysis in F-T mixtures has shown that both the reaction rates and enantioselectivities are controlled by pH and are affected by the F/T molar ratio. Likewise, many studies have utilized the azeotropic mixture of formic acid (HCOOH) and triethylamine (NEt_3_) (F-T), with a F/T molar ratio of 2.5:1 [81,82,83]. Ashutosh A. Kelkar et al. investigated the asymmetric transfer hydrogenation of imines in water by varying the formic acid to triethylamine ratio, finding that the optimum ratio was 1.1, using Rh-(1*S*,2*S*)-TsDPEN as a catalyst (Figure 3) [84].

Research in modern chemistry has focused heavily on catalysis in water due to water’s role as an eco-friendly solvent [85,86]. In this context, the asymmetric transfer hydrogenation (ATH) of ketones has been investigated in water with F/T being the hydrogen source [87,88]. The reaction’s efficiency and enantioselectivity are affected by the F/T ratio. For metal-catalyzed ATH of imines, the F/T molar ratio is typically 5:2 when water is the solvent [89]. The addition of methanol as a cosolvent enhances the reaction by forming hydrogen bonds and disrupting the strong hydrogen bonding in pure water, making reactants/products more soluble in the reaction mixture [84,90,91].

The main drawback of using azeotropic F-T for ATH is that certain catalysts undergo fast decomposition or lose their catalytic activity [15]. Additionally, there might be a prolonged delay before the reaction starts, leading to a long reaction time needed to achieve high conversion [87].

Erick M. Carreira et al. carried out an asymmetric transfer hydrogenation of β-keto esters in water, employing formic acid/sodium formate as the hydrogen source [92]. This reaction was performed in an open-air environment and resulted in high yields and selectivity of β-hydroxy esters.

This process is compatible with substrates containing electron-donating and -withdrawing groups in the *para*- and *meta*-positions. Notably, variations in pH had a minimal impact on the reaction’s enantioselectivity. Excellent enantiomeric excess was maintained within a pH range of 3.5 to 10.0, achieving a complete conversion (Table 4).

Suzzane Chayya et al. observed that the presence of a base significantly impacts the resulting products in the catalytic transfer hydrogenation of 4-(phenylethynl) acetophenone. Under the conditions of utilizing 2% Pd(acac)_2_ as a catalyst for 24 h at 80 °C, two different products are obtained based on variations in the (Hydrogen Donor/Base) condition (Table 5) [93], product **B** [(*E*)-1-(4-styrylphenyl) ethenone], and product **C**, which is an acetylphenanthrene derivative, namely [1-(phenanthrene-3-yl)] ethenone]. Product **C** is formed through electrocyclic ring closure at thermal conditions of 60 °C, involving [(*Z*)-1-(4-styrylphenyl) ethenone], which was not isolated as a separate compound.

The reduction reaction occurs specifically at the alkyne level, resulting in the formation of an alkene, while the carbonyl group remains unaffected. A similar selective reduction pattern was identified in their study of 4-phenyl-3-butyene-2-one, where the reduction of the alkyne resulted in the formation of both the corresponding alkene and alkane (Table 6) [94].

**Scheme 12:**
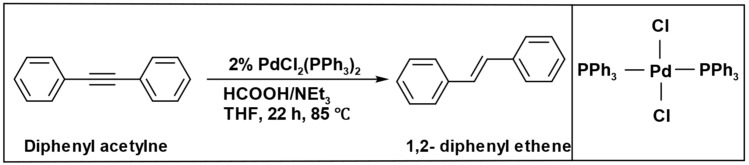
Catalytic transfer hydrogenation of diphenyl acetylene using a palladium catalyst and formic acid as the hydrogen source (Chayya et al., 2021) [94].

The formation of the alkane was not observed when diphenyl acetylene was subjected to prolonged high-temperature conditions during the reduction process. Instead, under the same operational parameters, only (1,2-diphenyl ethene) was produced (Scheme 12) [94].

Formic acid has been utilized as a hydrogen donor in numerous catalytic reactions, often without the need for additional base additives. Matthias Beller et al. performed different studies using Fe (BF_4_)_2_·6H_2_O combined with tetraphosphorus as a catalyst. The catalyst demonstrated excellent efficiency in the selective transfer hydrogenation of a broad spectrum of aromatic and aliphatic compounds, including alkyne [95], and nitro compounds [96]. The catalytic transfer hydrogenation reactions were done in the absence of base additive.

Interestingly, iron catalysts have gained significant attention due to their affordability, wide availability, and low toxicity. This has driven research efforts towards substituting precious metals with iron-based alternatives [97].

**Scheme 13:**
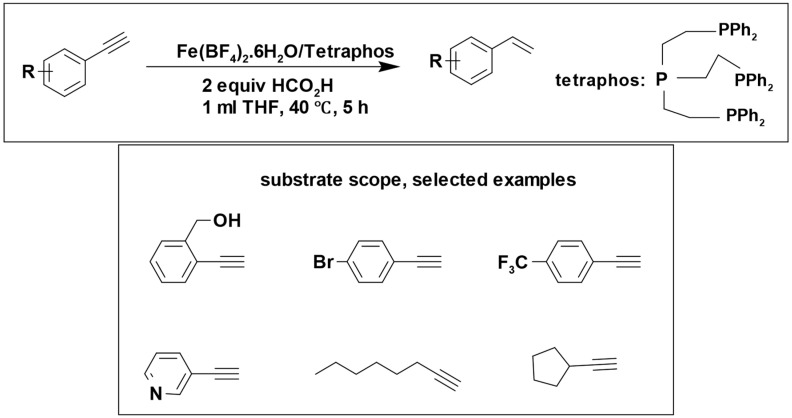
Catalytic transfer hydrogenation of an alkyne using an iron catalyst and formic acid as the hydrogen source (Wienhöfer et al., 2012) [95].

The reduction of alkynes to alkenes was successfully accomplished with a remarkable yield of 99%, and the reaction demonstrated great tolerance to a variety of functional groups (Scheme 13) [95].

**Scheme 14:**
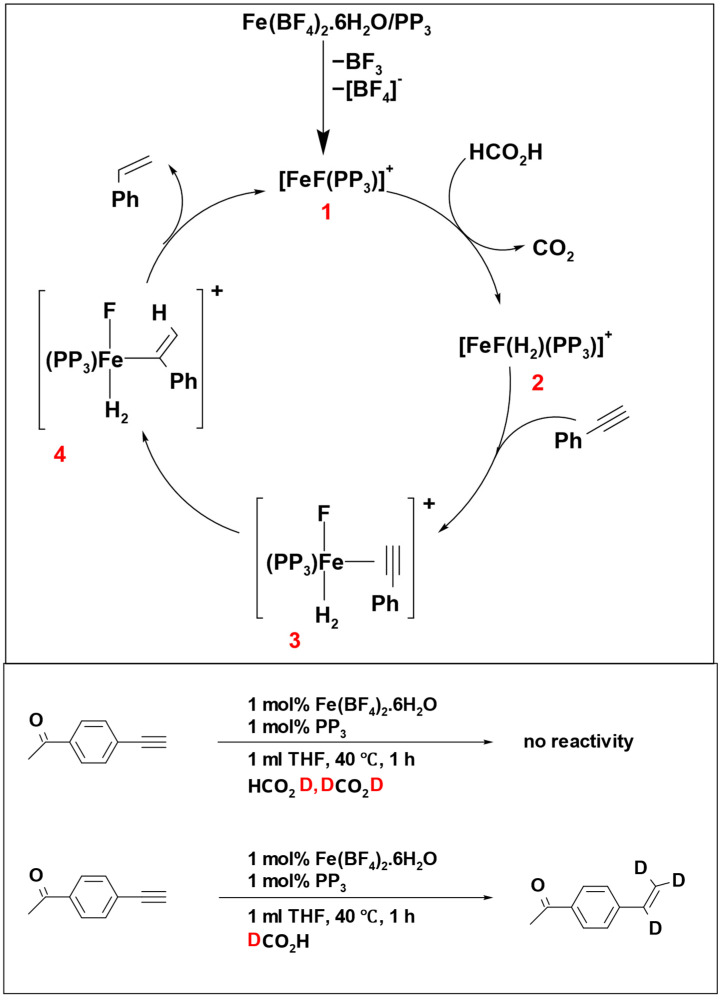
Catalytic cycle of iron-catalyzed transfer hydrogenation of an alkyne using formic acid as the hydrogen source (Wienhöfer et al., 2012) [95].

The reaction occurred in four steps (Scheme 14). In the first step, ligand PP3 coordinates with the iron precursor to form the catalytically active species (1). In step 2, the active complex breaks down the formic acid, releasing carbon dioxide to generate [FeF(H_2_)(PPh_3_)]^+^ (2). In step 3, phenylacetylene binds to the iron center in complex **3** and gets reduced to an alkene in complex **4**. Finally, styrene is obtained, and the original state of catalyst **1** is regenerated. The most important step involves the coordination and activation of the carboxylic acid group by the active [FeF(PPh_3_)]^+^ species. This was confirmed through experiments involving various deuterated formic acids (HCO_2_D, DCO_2_D, and DCO_2_H), revealing reactivity only in the formic acid with deuterium attached to the carbon atom.

**Scheme 15:**
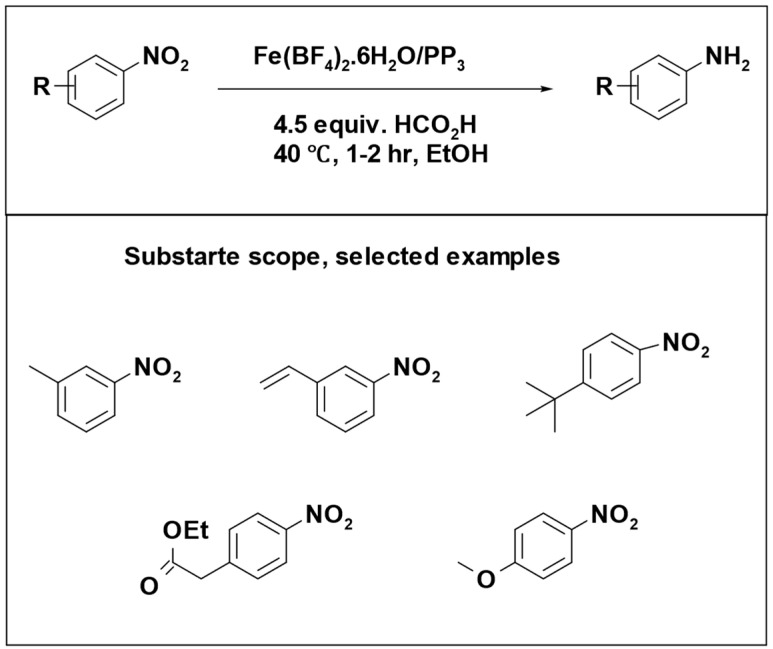
Catalytic transfer hydrogenation of nitro compounds using an iron catalyst and formic acid as the hydrogen source (Wienhöfer et al., 2011) [96].

The conversion of nitro compounds into anilines was successfully accomplished, resulting in high yields. A wide range of substrates with different functional groups were effectively converted. Importantly, this represented the first catalytic transfer hydrogenation of nitro compounds without the need for a base (Scheme 15) [96].

**Scheme 16:**
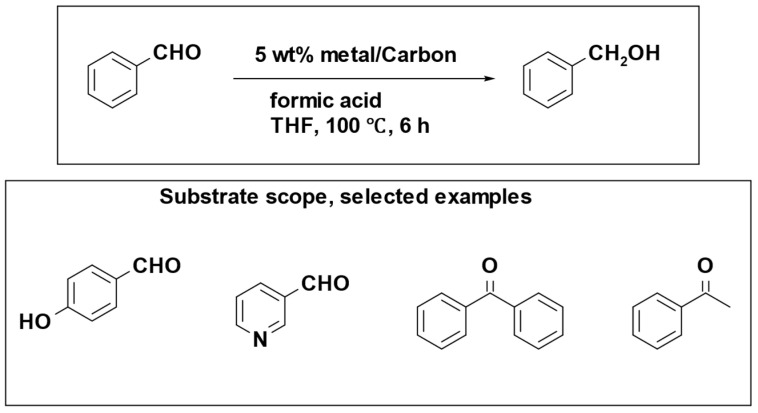
Catalytic transfer hydrogenation of benzonitrile using a palladium-supported catalyst in the presence of formic acid as a hydrogen donor (Tomar et al., 2020) [98].

The iron catalyst showed exceptional performance in both its activity and selectivity. However, a notable drawback was observed in terms of catalyst reusability. In light of this, researchers explored an alternative approach through the development of a reusable heterogeneous catalyst. Kohki Ebitani et al. conducted a study involving a supported palladium catalyst for the reduction of aromatic carbonyl compounds, utilizing formic acid as a hydrogen source under mild reaction conditions (Scheme 16). The most efficient support materials were found to be carbon and alumina, providing optimal results for the reduction of benzaldehyde and benzonitrile, respectively [98].

The catalytic system is not efficient in reducing aliphatic and carbonyl compounds containing heteroatoms, which is attributed to the low electron density of the carbonyl carbons and the strong interaction of heteroatoms with the active Pd sites.

Furthermore, the reusability of the Pd/Al_2_O_3_ catalyst was assessed in the reduction of benzonitrile using formic acid. The results indicated that the recovered catalyst could be recycled up to three times without a significant decrease in product yield and selectivity.

Similarly, several catalytic transfer hydrogenation reactions have been conducted using formic acid as a hydrogen source in combination with reusable supported catalysts under base-free conditions [99,100].

**Scheme 17:**
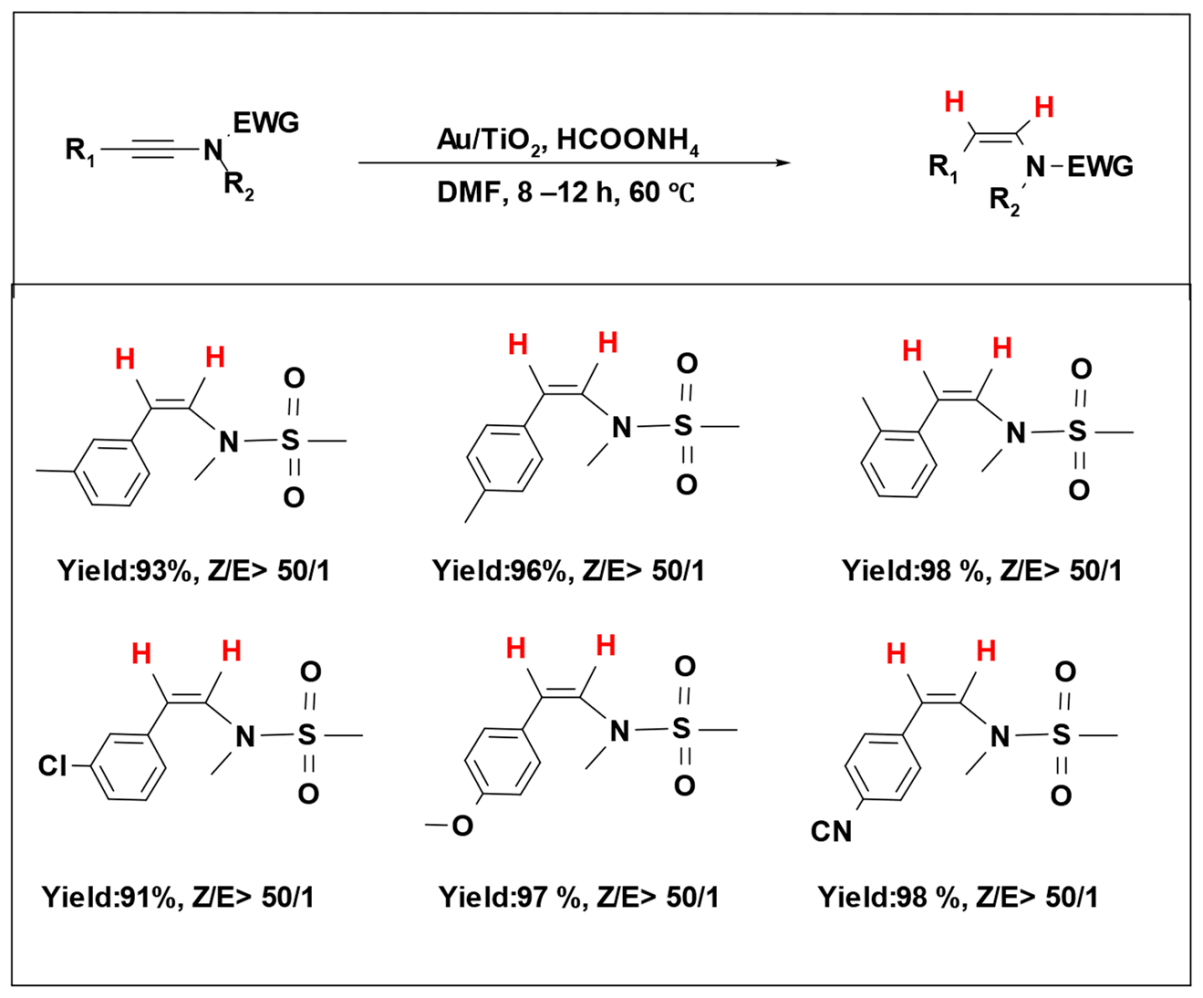
Catalytic transfer hydrogenation of ynamides using a gold catalyst in the presence of ammonium formate as a hydrogen source (Lin et al., 2019) [101].

Formic acid’s inherent acidity poses challenges when used for carbonyl compound reduction due to the strong interactions between the hydrogen donor and the catalytic system, leading to the inhibition or even degradation of the catalytic species [78]. To overcome this, researchers have studied the utilization of formic acid salts as hydrogen donor molecules in catalytic hydrogenation reactions. Ammonium formate, an easily manageable and cost-effective hydrogen source, has been combined with a gold catalyst to perform a selective hydrogenation of ynamides, yielding Z-enamides (Scheme 17). Notably, the catalyst maintained its performance over five reaction cycles, showing excellent catalytic activity and selectivity [101].

**Scheme 18:**
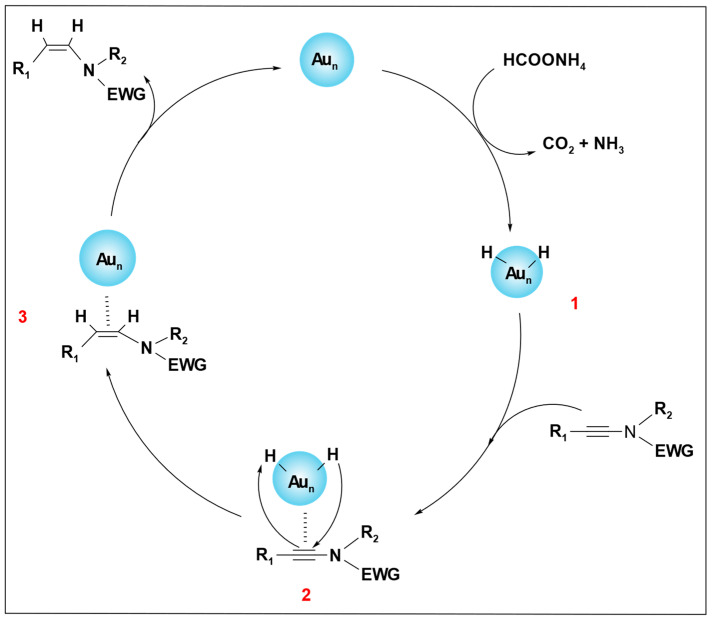
Catalytic cycle of the transfer hydrogenation of ynamides using a gold catalyst and ammonium formate as a hydrogen source (Lin et al., 2019) [101].

The catalytic mechanism, as depicted in Scheme 17, begins with the breakdown of ammonium formate over the Au catalyst, which produces a hydrogen atom. This hydrogen atom adsorbs on the surface of the Au nanoparticles. A partially charged intermediate, Au–H (**1**)**,** is produced after the dissociation of the hydrogen, which coordinates with the C≡C bond to form complex (**2**). A hydrogen transfer occurs from complex (**2**) to the C≡C bond via *cis* addition. In complex (**3**), the *cis* addition product (alkene) is formed and dissociates from the surface of the Au, regenerating the active Au(0) catalyst (Scheme 18).

**Scheme 19:**
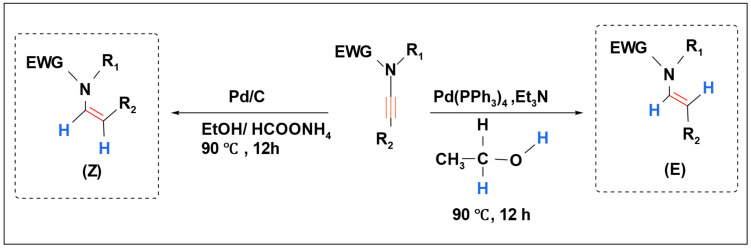
Stereoselective hydrogenation of ynamides using palladium as a catalyst and HCOONH_4_/EtOH and EtOH as hydrogen sources (Siva Reddy & Kumara Swamy, 2017) [102].

K. C. Kumara Swamy et al. investigated the stereoselective hydrogenation of ynamides using a palladium catalyst. They employed two hydrogen sources, HCOONH_4_/EtOH and EtOH, resulting in the formation of *E*-enamides and *Z*-enamides, respectively (Scheme 19) [102].

Eric V. Johnston et al. conducted a catalytic hydrogenation reaction for reduction of 4-nitroanisole to 4-methoxyaniline. They utilized Pd(0)-AmP-MCF as the catalyst and examined various hydrogen donors, including formic acid and formate salts. The most optimal result, with a yield exceeding 99%, was obtained using cesium formate salt as the hydrogen source (Table 7) [103].

### 2.4. 1,4-Cyclohexadiene

Although the use of 1,4-cyclohexadiene as a hydrogen donor in catalytic transfer hydrogenation is not common, its application has shown a complete conversion with a good yield.

John F. Quinn and his team achieved efficient catalytic transfer hydrogenation of heteroaromatic nitro groups [104] by using 1,4-cyclohexadiene as a hydrogen donor under microwave heating. Notably, microwave heating technology serves as a valuable alternative to high-temperature and high-pressure conditions, allowing for rapid and safe reactions [105]. This technique has been employed in various catalytic transfer hydrogenation processes [106,107].

**Scheme 20:**
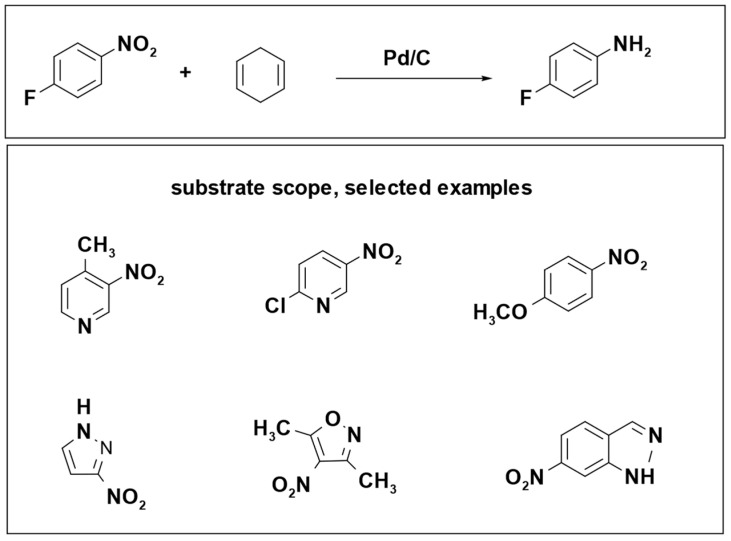
Catalytic transfer hydrogenation of 4-fluoronitrobenzene using a palladium-supported catalyst in the presence of 1,4-cyclohexadiene as a hydrogen donor (Quinn et al., 2010) [104].

The reduction of 4-fluoronitrobenzene led to a remarkable yield exceeding 99%, and the reaction produced only benzene as a byproduct, which can be easily separated and removed from the product mixture. The optimal conditions for this reaction involved the use of a Pd/C catalyst, six equivalents of 1,4-cyclohexadiene, microwave heating at 120 °C, and methanol as the solvent, with a reaction time of 5 min (Scheme 20) [104].

The reduction of cinnamic acid using a Pd/C catalyst and 1,4-cyclohexadiene as the hydrogen source selectively yielded 3-phenylpropionic acid [108]. The outcome of the reaction was notably influenced by both the quantity of 1,4-cyclohexadiene equivalents and the temperature at which the reaction was conducted (Table 8). When employing 2 equivalents of 1,4-cyclohexadiene (as indicated in entry 1), only a minimal 2% conversion of cinnamic acid was detected. However, employing 3 or more equivalents led to conversions exceeding 95% (Entries 2, 3, 4). Entries (6, 7, 8) indicated that catalytic transfer hydrogenation does not occur below 80 °C. Substituting the hydrogen donor with cyclohexene or replacing the Pd catalyst with Pt catalyst resulted in no reaction.

In the process of peptide synthesis, catalytic transfer hydrogenation plays a vital role by eliminating protective groups that prevent amino acid polymerization and reduce undesired side reactions [109]. Among these protective strategies, the benzyl group is commonly employed [110,111,112]. In this context, Arthur M. Felix et al. effectively eliminated the *N*-benzyloxycarbonyl protective group from L-alanine using a catalytic transfer hydrogenation technique. Their method relied on the use of a 10% Pd/C catalyst and 1,4-cyclohexadiene as the hydrogen donor (Table 9) [113].

### 2.5. Glycerol

Glycerol has gained attention as a green solvent in various organic reactions and synthetic processes [114,115]. It is considered non-hazardous and possesses unique physical and chemical properties that enable the dissolution of acids, bases, transition metal complexes, inorganic compounds, and even organic compounds that have limited solubility in water. Notably, glycerol’s high boiling point allows for efficient separation of products using a simple distillation technique.

**Scheme 21:**
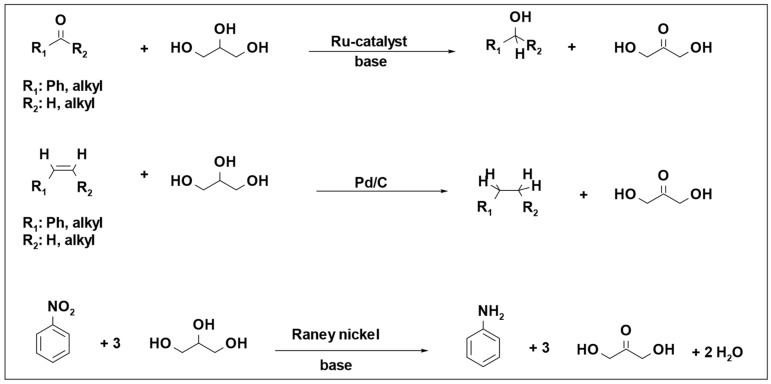
Catalytic transfer hydrogenation of benzaldehyde, nitrobenzene, and styrene, using glycerol as the hydrogen source (Wolfson et al., 2009) [116].

Remarkably, glycerol has proven effective as a hydrogen donor in catalytic transfer hydrogenation reactions involving various unsaturated organic compounds. Adi Wolfson et al. explored the reduction of benzaldehyde, nitrobenzene, and styrene, using different catalysts and glycerol as the hydrogen source (Scheme 21) [116].

The reduction of benzaldehyde and benzene requires the presence of a base. In contrast, the catalytic transfer hydrogenation of styrene should be conducted without adding a base, as introducing a catalyst in the presence of a base deactivates the catalyst. This is due to the higher oxidation potential of the secondary alcohol, which causes glycerol to dehydrogenate into dihydroxyacetone. Subsequently, dihydroxyacetone decomposes under the reaction conditions.

**Scheme 22:**
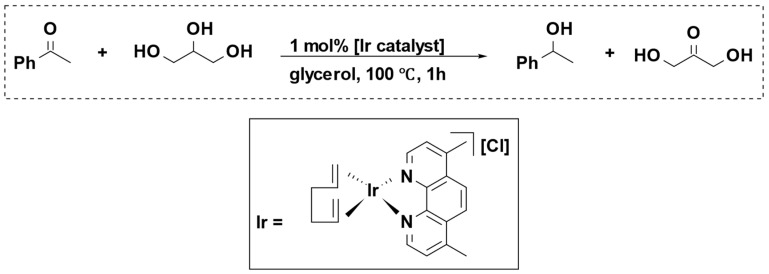
Transfer hydrogenation reaction in the presence of an iridium catalyst, using glycerol as a hydrogen donor (Farnetti et al., 2009) [118].

Numerous studies have highlighted the utility of glycerol as both a solvent and a reducing agent in the synthesis of nanoparticles and metal-catalyzed transfer hydrogenation reactions [117]. Farnetti et al. identified iridium catalyst as particularly effective for reducing acetophenone, using glycerol as a hydrogen donor (Scheme 22) [118].

Furthermore, iridium (III and I) catalysts were also investigated for the reduction of carbonyl compounds, namely benzaldehyde and acetophenone, using glycerol as a hydrogen donor. After heating for 7 h, the desired alcohol products were obtained in high yields [119,120].

Having mentioned above the most frequently used hydrogen donor in the catalytic transfer hydrogenation, it is important to shed the light on the remarkable studies carried out by Hai-Xu Wang et al. [121]. Different from the homogeneous counterparts, they performed an electrocatalytic hydrogen reduction reaction where hydrogen gas (H_2_) was used to generate reactive hydride species directly on the surface of platinum electrodes. These reactive hydrides can then be transferred to hydride acceptors molecules, enabling the hydrogenation reaction of different unsaturated compounds.

In a new perspective, Hua Aun et al. successfully performed the hydrogenation of 4-nitrophenol in an aqueous phase using a supported metal catalyst through an Electron-Proton Transfer Mechanism. Their approach offers a practical way to distinguish between the proton-electron transfer pathway and the hydrogen-atom transfer pathway in hydrogenation [122]. This important approach has the potential to be applied to a wide range of reaction types, paving the way for further research and development in the field of hydrogenation.

These extensive studies and the development in the catalytic transfer hydrogenation reaction reflect its high importance. It has diverse applications in different industries, leading to the production of valuable products. In the pharmaceutical sector, it plays a vital role because many pharmaceutical drugs contain stereocenters, which are crucial for their bioactivity. Thus, the production of single enantiomer drugs is vital. One effective method for achieving this is utilizing asymmetric transfer hydrogenation of ketones to selectively produce one enantiomeric secondary alcohol.

In addition to its role in pharmaceuticals, it has also gained recognition in the chemical industry. This is exemplified by its application in the conversion of furfural to furfuryl alcohol or tetrahydrofurfuryl alcohol which serves as a fundamental raw material for the synthesis of various high-value chemicals, including diols, dihydropyran, pyridine, and numerous others [123].

Moreover, catalytic transfer hydrogenation is indispensable in the food industry. It is utilized to transform liquid vegetable oils into solid fats, enhancing not only the texture of food products but also prolonging their shelf life. This property is particularly valuable in the production of items such as margarine and other solid, fat-based foods [124].

In summary, this reaction is a critical chemical process with a wide range of industrial applications, spanning chemicals, pharmaceuticals, and food. It serves the purpose of producing specific chemical compounds and improving product characteristics.

## 3. Future Trends

In our exploration of catalytic transfer hydrogenation reactions, we have highlighted the primary works and findings in both the academic and industrial sectors. Catalytic transfer hydrogenation methods have emerged in the world of chemical reactions with distinct benefits over the traditional approach, such as using safer, more readily available, and more cost-effective hydrogen sources, and enabling the recycling of byproducts. Considering these distinctive characteristics, there are numerous potential future trends for investigating and expanding the scope of catalytic transfer hydrogenation.

### 3.1. Biomass-Derived Hydrogen Donors

Biomass feedstock such as agricultural residues and waste biomass can be converted, through a fermentation process, to bioalcohols such as ethanol and butanol. These molecules have demonstrated their potential as effective hydrogen donors in catalytic transfer hydrogenation. This approach carries significant environmental implications, contributing positively to the circular economy by upcycling and repurposing biomass waste streams.

### 3.2. Hydrogen Storage Material

Hydrogen storage material can provide a new aspect of transfer hydrogenation reaction. Among these materials, metal-organic frameworks (MOF) are characterized by their porous structure and high surface area. Such properties offer a safe and controlled approach to hydrogen storage and release for catalytic transfer hydrogenation reactions.

### 3.3. Catalyst Development

Catalyst development aims to create better catalysts by exploring new metal complexes or catalytic systems with improved catalytic activity, selectivity, and recyclability. Additionally, catalyst design helps enhance catalyst properties, such as active sites, in order to achieve optimal performance in catalytic transfer hydrogenation reactions.

Moreover, the ongoing advancement of chiral catalysts will further facilitate asymmetric transfer hydrogenation, enabling the selective synthesis of chiral compounds. These chiral compounds hold particular importance in the pharmaceutical and agrochemical industries.

Through thorough investigation, the synergistic effect between a catalyst and a particular hydrogen donor can be revealed. This insight carries significant implications as it allows for control over the kinetics of reactions and their outcomes. Such control plays a vital role, especially in industries where the efficient formation of specific compound is crucial.

### 3.4. Green Solvents

The choice of solvent is crucial in catalytic transfer hydrogenation processes. Environmentally friendly solvents such as glycerol and ionic liquids are promising alternatives to standard volatile organic solvents. Recent studies have shown that glycerol can serve both as a green solvent and a hydrogen source, which is a significant advancement. Looking ahead, a possible future trend could involve further research involving glycerol in a wider range of catalytic transfer hydrogenation reactions.

Ionic liquids, on the other hand, have unique characteristics, including low volatility and high thermal stability, making them excellent green solvents for various chemical processes. Their eco-friendliness, attributed to their non-toxic nature and reduced environmental impact, makes them as attractive replacements for traditional organic solvents.

### 3.5. Heating Methods

In catalytic transfer hydrogenation, the choice of heating method plays a critical role in influencing the outcomes of reactions. Typically, conventional heating methods have been used, alongside microwave heating, in a few studies. However, considering the variety of available heating methods, exploring these newer approaches holds promise for the future.

Infrared heating is a preferable alternative to conventional heating. While both methods primarily heat surfaces, infrared heating offers advantages including rapid, direct, and uniform heat distribution, making it an efficient choice. Those characteristics render it a good selection for catalytic transfer hydrogenation, particularly in applications requiring precise temperature control and high temperature gradients.

Ultrasound-assisted heating uses ultrasonic waves to create localized heating within a reaction mixture. This approach can facilitate reactions, promising improved outcomes and resource efficiency.

Photothermal heating involves using materials such as nanoparticles, capable of absorbing light energy to generate localized heat. It is a potentially sustainable and efficient heating method, especially suitable for specific catalytic transfer hydrogenation reactions that require precise heating control.

Hybrid heating methods, as the name suggests, combine different heating techniques to provide unique advantages. For example, researchers might utilize microwave heating for quick and bulk heating of an object and then switch to conventional or infrared heating for precise temperature maintenance. Such hybrid approaches can improve selectivity and overall reaction efficiency.

### 3.6. Nature of Base

Bases have proven to be effective additives in catalytic transfer hydrogenation reactions, where they enhance catalytic activity by facilitating substrate deprotonation and its subsequent interaction with the metal ion. Organic bases such as amines (e.g., trimethylamine) are commonly employed in these reactions. Investigating different bases is a valuable path for future exploration.

Solid bases, such as alkaline metal oxides or hydroxides supported on porous materials such as zeolites, offer the advantage of easy separation and recyclability when used as a source of base. Additionally, there is potential to utilize green bases in catalytic transfer hydrogenation, such as amino acids or natural alkaloids. These environmentally friendly bases not only contribute to the catalytic process but also align with the principles of green chemistry.

These future trends represent exciting possibilities in catalytic transfer hydrogenation, promising advancements in sustainability, selectivity, and efficiency across various industries and applications.

## 4. Concluding Remarks

In conclusion, this article delves into the realm of catalytic transfer hydrogenation reactions, which have garnered attention in both academic and industrial sectors. The conventional approach of direct hydrogenation using pressurized hydrogen gas has been significantly challenged by the emergence of transfer hydrogenation methods. This innovation offers a safer and more accessible alternative, utilizing various hydrogen donors to efficiently reduce unsaturated compounds.

The exploration of potential hydrogen donors in transfer hydrogenation reactions has been a focus of this work. Notably, alcohols, formic acid, 1,4-cyclohexadiene, water, and glycerol have been investigated as versatile hydrogen sources. Each donor exhibits unique properties and characteristics that influence their compatibility with different catalysts, reaction conditions, and types of unsaturated compounds.

Alcohols, such as primary and secondary alcohols, play a crucial role in the Meerwein–Ponndorf–Verley (MPV) reduction and offer valuable hydrogen transfer capabilities. Primary alcohols, such as methanol and ethanol, have demonstrated efficacy in various reductions, while secondary alcohols, especially isopropanol, have revealed compatibility with diverse substrates and stability within catalytic systems. However, challenges related to reactivity and side reactions have led to the exploration of alternative hydrogen donors.

Formic acid and its salts stand out as efficient and irreversible hydrogen donors. Their ability to prevent reverse reactions and their compatibility with various catalysts make them attractive choices in catalytic transfer hydrogenation, particularly for producing chiral compounds.

Unconventional donors such as 1,4-cyclohexadiene and glycerol have shown excellent activity in the reduction of specific unsaturated compounds, with successful results obtained under specific conditions such as microwave heating. Glycerol, known for its green properties, also serves as an intriguing green solvent in these reactions.

In summary, this article has discussed the various hydrogen donor molecules used in catalytic transfer hydrogenation. It has also outlined prospective trends, including using biomass-based sources for hydrogen, exploring hydrogen storage materials such as the metal-organic framework, developing more efficient catalysts, and adopting eco-friendly solvents such as glycerol and ionic liquids. Additionally, innovative heating methods, diverse base materials, and continued research into catalyst-hydrogen donor interactions will shape the future of catalytic transfer hydrogenation, making it more sustainable and effective.

## Figures and Tables

**Table 1:** Iron-catalyzed hydrogen transfer reduction of methyl benzoate in the presence of various alcohols as hydrogen donors (Farrar-Tobar et al., 2018) [26].

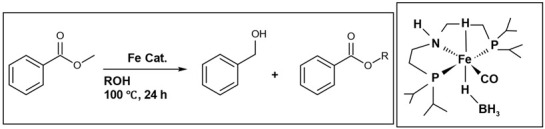					
Entry	Cat. Load	Hydrogen Source	Conv. [%]	% Yield of b	% Yield of c
1	5	EtOH	>99	88	10
2	5	MeOH	7	0	0
3	5	iPrOH	95	56	39
4	5	BuOH	>99	40	60
5	3	EtOH/MeOH	98	61	35

**Table 2:** Transfer hydrogenation reaction of furfural using an iron catalyst with various hydrogen donors (Valekar et al., 2020) [54].

Entry	Hydrogen Donor	Conversion [%]	Yield [%]	Selectivity [%]
1	methanol	7.8	0.5	6.4
2	ethanol	24.3	11.7	48.1
3	1-propanol	19.7	7.2	36.5
4	2-propanol	51.5	44	85.4
5	2-butanol	71.3	60	84.2
6	cyclohexanol	19.5	1.6	8.2

**Table 3:** Catalytic transfer hydrogenation of furfural using various catalysts and hydrogen donors.

Entry	Catalyst	Hydrogen Source	% Yield	Ref.
1	Cu-Al	methanol	99	[56] (Zhang & Chen, 2017)
2	Cu/MgO-Al_2_O_3_	isopropanol	89	[57] (Chen et al., 2018)
3	Ni-Fe_2_O_4_	isopropanol	94	[58] (He et al., 2018).
4	ZrHAs	isopropanol	97	[59] (Sha et al., 2017)
5	Php-zr	isopropanol	78	[60] (Li et al., 2019)
6	Al_2_O_3_-S	isopropanol	96	[61] (Kim et al., 2017)
7	Pd/NPC	2-butanol	90	[62] (Puthiaraj et al., 2019).
8	MgO	methanol	58	[63] (Grazia et al., 2016)
9	Al_7_Zr_3_@Fe_3_O_4_	isopropanol	90.5	[64] (He et al., 2017).
10	γ-Fe_2_O_3_@HAP	isopropanol	91.7	[65] (Wang & Zhang, 2016)

**Table 4:** A pH-independent asymmetric transfer hydrogenation of β-keto esters in water using an iridium catalyst with formic acid/sodium formate as the hydrogen donor (Ariger & Carreira, 2012) [92].

Entry	R	pH	Yield %	ee
1	C_6_H_5_	8	96	96
2	C_6_H_5_	3.5	98	92
3	*p*-F-C_6_H_4_	8	95	94
4	*p*-OMe-C_6_H_4_	8	97	95
5	*O*-Cl-C_6_H_4_	8	97	70
6	3-pyridyl	8	77	73
7	2-furyl	8	90	96

**Table 5:** Catalytic transfer hydrogenation of 4-(phenylethynl) acetophenone using a palladium catalyst with various (H-Donor/Base) molecules (Chayya et al., 2022) [93].

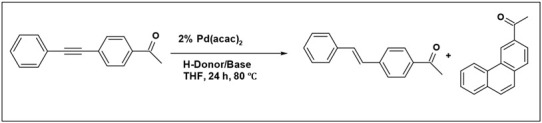			
Entry	H-Donor/Base	Ratio % of B	Ratio % of C
1	HCOOH/NEt_3_	0	100
2	HCOOH/K_2_CO_3_	100	0
3	iPrOH/NEt_3_	0	100
4	DMF/KOH	64	36
5	DMF/NEt_3_	0	100

**Table 6:** Catalytic transfer hydrogenation of 4-(phenylethynl) acetophenone using a palladium catalyst and formic acid as a hydrogen donor at different temperatures and times (Chayya et al., 2021) [94].

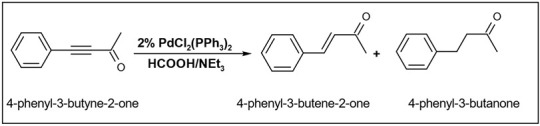				
Entry	Time	T (°C)	% (4-Phenyl-3-butene-2-one)	%(4-Phenyl-3-butanone)
1	4	50	100	0
2	22	50	90	10
3	22	85	28	72

**Table 7:** Catalytic hydrogenation reaction of 4-nitroanisole using various hydrogen donors (Verho et al., 2013) [103].

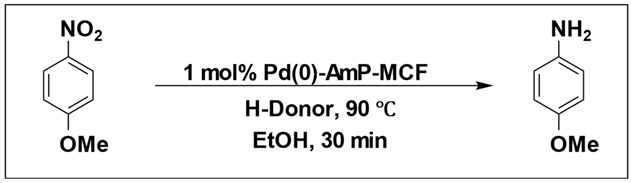			
Entry	H-Donor	Equiv.	% Yield
1	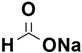	3	73
2	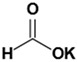	3	91
3	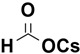	3	>99
4	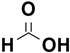	11	14
5	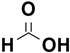	Neat	0
6	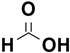	3	85

**Table 8:** Catalytic transfer hydrogenation of cinnamic acid using a Pd/C catalyst and 1,4-cyclohexadiene as the hydrogen source (Quinn et al., 2008) [108].

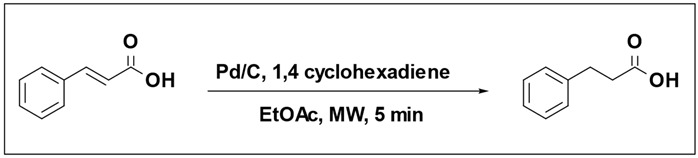			
Entry	Cyclohexadiene (Equiv.)	Temperature	% Yield
1	2	100	2
2	3	100	95
3	4	100	94
4	5	100	>99
6	5	60	0
7	5	70	0
8	5	80	96

**Table 9:** Catalytic transfer hydrogenation reaction using 1,4-cyclohexadiene as the hydrogen donor and 10% Pd/C catalyst for the removal of the *N*-benzyloxycarbonyl protecting group (Felix et al., 1978) [113].

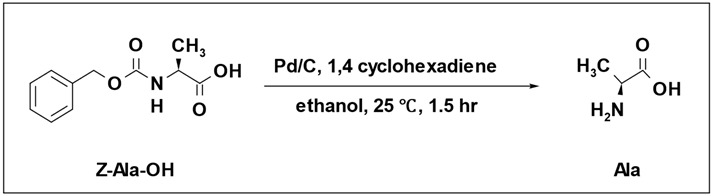		
Substrate	Product	% Yield
*Z*-Ala-OH	Ala	95
Boc-Lys(*Z*)-OH	Boc-Lys-OH	88
*Z*-Ser-OBz	Ser	99
Boc-Tyr(Bz)-OH	Boc-Tyr-OH	100
*Z*-Phe-OH	Phe	99

## Data Availability

Not applicable.
